# Meat Consumption and Cancer Risk

**DOI:** 10.1371/journal.pmed.0040345

**Published:** 2007-12-11

**Authors:** Jeanine M Genkinger, Anita Koushik

## Abstract

The authors review the key studies on the association between meat intake and cancer risk, including a new prospective cohort study by Amanda Cross and colleagues published in*PLoS Medicine.*

The large international variation in incidence rates of cancer, together with findings from migrant studies, suggest that environmental factors such as diet are associated with cancer risk. The intake of meats, such as beef, varies 3-fold across the world—consumption is highest in developed countries (23 kg/capita) compared to less developed countries (6 kg/capita) [1]. Based on Richard Doll and Richard Peto's work in 1981, it has been estimated that approximately 35% (range 10%–70%) of cancer can be attributed to diet, similar in magnitude to the contribution of smoking to cancer (30%, range 25%–40%) [2].

Meat consumption in relation to cancer risk has been reported in over a hundred epidemiological studies from many countries with diverse diets. The association between meat intake and cancer risk has been evaluated by looking both at broad groupings of total meat intake, and also at finer categorizations, particularly intakes of red meat, which includes beef, lamb, pork, and veal, and also more specifically processed meats, which includes meats preserved by salting, smoking, or curing.

Although the association of cancer and meat intake may be partially explained by high-energy or high-fat (“westernized”) diets, of greater interest is a possible direct role of potentially carcinogenic compounds that are found in meats, including N-nitroso compounds, heterocyclic amines, or polycyclic aromatic hydrocarbons. N-nitroso compounds are broad-acting potent carcinogens in animal models [3] and include nitrosamines, which require metabolic activation to be converted to a carcinogenic form, and nitrosamides, which do not require activation. Similarly, heterocyclic amines are classified as mutagens and animal carcinogens [4–8]. These compounds and others present in meats (salts, nitrates, nitrites, heme iron, saturated fat, estradiol) have been theorized to increase DNA synthesis and cell proliferation, increase insulin-like growth factors, affect hormone metabolism, promote free radical damage, and produce carcinogenic heterocyclic amines [9–16], all of which may promote the development of cancer.

**Linked Research Article**

This Research in Translation article discusses the following new study published in *PLoS Medicine:*

Cross AJ, Leitzmann MF, Gail MH, Hollenbeck AR, Schatzkin A, et al. (2007) A prospective study of red and processed meat intake in relation to cancer risk. PLoS Med 4(12): e325. doi:10.1371/journal.pmed.0040325

Using data from a large cohort study, Amanda Cross and colleagues found that both red and processed meat intakes were positively associated with cancers of the colorectum and lung.

## Colorectal Cancer

The malignancy most extensively studied in relation to meat intake has been colorectal cancer. In ecological studies, correlations between international per capita meat intakes and colon cancer incidence (*r* > 0.85) and mortality (*r* > 0.70) rates have been high [17,18]. Similarly, raised colorectal cancer risks in relation to both red and processed meat intakes have been observed in case-control and cohort studies. A 1997 review of these studies, sponsored by the World Cancer Research Fund and the American Institute for Cancer Research, concluded that the intake of red meat *probably* increases the risk of colorectal cancer, while processed meat *possibly* increases colorectal cancer risk [19]. A similar consensus was reported by the Colon Cancer Panel at the World Health Organization consensus conference [20] and the Working Group on Diet and Cancer of the Committee on Medical Aspects of Food and Nutrition Policy [21]. In recent meta-analyses of colorectal cancer that included studies published up to 2005 [22–24], summary associations indicated that red meat intakes were associated with 28%–35% increased risks while processed meats were associated with elevated risks of 20%–49%.

## Other Types of Cancer

Additionally, a large number of studies have examined the association between meat intake and stomach cancer risk. In a recent meta-analysis, positive associations were observed between processed meat consumption and stomach cancer risk, although the results from case-control versus cohort studies were heterogeneous [25]. Fewer studies with less consistent associations have been reported for cancers of the bladder [26,27], breast [28,29], endometrium [30], glioma [31], pancreas [32–34], prostate [35], and renal cell [36]. There has been even less research on the association between meat intake and cancers of the lung [37,38], esophagus [39], oral cavity [40,41], ovary [42–44], cervix [45], and liver [41]. Most of the studies examining these sites have been case-control, and some of the earlier studies lacked adjustment for energy intake or body mass index, two key potential confounders.

## A New Study of Multiple Cancer Sites

In this issue of *PLoS Medicine*, Amanda Cross and colleagues present their findings from a large prospective cohort study on the association between red and processed meat intake and cancer at several sites [46]. Their analysis is based on the prospective National Institutes of Health (NIH)-AARP (formerly known as the American Association of Retired Persons) Diet and Health Study and includes almost 500,000 men and women in the United States, among whom over 53,000 incident cancers occurred.

For colorectal cancer, a 24% increased risk with red meat consumption of 62.5 g/1,000 kcal and a 20% increased risk with processed meat consumption of 22.6 g/1,000 kcal was observed among both men and women, which is similar in magnitude to the summary relative risks observed in previous meta-analyses [22–24]. The researchers also found that increasing intakes of red meat were significantly associated with elevated risks of 20%–60% for cancers of the esophagus, liver, and lung. For processed meats, a 16% increased risk of lung cancer was observed. Red and processed meat intake was associated with an increased pancreatic cancer risk in men only.

The results from the NIH-AARP Diet and Health Study corroborate previous findings for colorectal cancer. However, a positive association with stomach cancer, which has been seen mostly in previous case-control investigations, was not observed in the NIH-AARP Diet and Health Study. Relative risks for stomach cancer have been primarily null in previous cohort studies compared to case-control studies, and thus the NIH-AARP Diet and Health Study results for stomach cancer are consistent with previous cohort studies.

**Five Key Papers in the Field**

**Larsson et al, 2006** [22] A meta-analysis of epidemiological studies of meat and colorectal cancer that used a prospective design, which is less susceptible to recall and selection biases.

**Larsson et al., 2006** [25] A thorough qualitative and quantitative review of case-control and cohort studies on stomach cancer, with a focus on intake of processed meat, which may contain higher levels of carcinogenic compounds compared to other meats.

**Missmer et al., 2002** [28] In this study, meat intake and breast cancer risk were examined by pooling the original primary data from eight prospective cohort studies, allowing for the analysis of various exposures and population subgroups. A pooled analysis is less susceptible to publication bias compared to meta-analyses of the published literature.

**Sinha R (2002) An epidemiologic approach to studying heterocyclic amines. Mutat Res 506–507: 197–204.** This article describes the development of a database to estimate intakes of heterocyclic amines, a potentially important contributor to the association between meat and cancer risk, from food frequency questionnaire data.

**Sinha R, Norat T (2002) Meat cooking and cancer risk. IARC Sci Publ 156: 181–186.** A general overview of the association between meat and cancer risk, with a special emphasis on cooking techniques.

In Cross and colleagues' study, higher consumption of meat was positively associated with risk of cancer of the lung, liver, esophagus, and pancreas, similar to the findings from some [32,37–40,47–52] but not all [33,41,53–58], previous case-control and cohort studies. An inverse association with endometrial cancer was observed in the NIH-AARP Diet and Health Study, which is in contrast to the positive association reported in the recent meta-analysis by Elisa Bandera et al. [30]. This meta-analysis was based on 16 case-control studies, among which recall and selection biases cannot be ruled out.

## Strengths and Weaknesses of the New Study

The NIH-AARP investigation is based on high-quality prospective dietary information obtained using a validated food frequency questionnaire (FFQ) with 124 items [59]. The analyses were conducted using only baseline FFQ data covering recent intakes; thus, changes in intakes of meat and other nutrients over time, as well as lifetime consumption patterns, could not be evaluated in the NIH-AARP study. Additionally, because the NIH-AARP study measured adult red and processed meat intake, it may not have captured the relevant exposure time for carcinogenesis, which may have occurred in childhood, adolescence, or early adulthood.

Nonetheless, in this study, diet was measured prior to diagnosis of cancer; thus, a cancer diagnosis would not have influenced the reporting of meat intake, minimizing the potential for recall bias. Additionally, the potential for selection bias was minimized as the cohort follow-up rate was very high (more than 95%). Furthermore, the NIH-AARP investigation is based on high-quality prospective measurement of other important environmental factors (e.g., smoking, body mass index), a long follow-up time (8.2 years), and a large number of cases of cancer. Because of the large population size, the NIH-AARP Diet and Health Study was able to prospectively analyze several rare cancer sites, including brain, laryngeal, non-Hodgkin lymphoma, pancreatic, pharyngeal, renal, and thyroid. The great variation in red and processed meat intake among the NIH-AARP population allowed for the examination of these specific cancer sites to be conducted with relatively sufficient power, thus greatly adding to the dearth of prospective literature to date on these rare cancers.

In interpreting the findings from studies of meat intake and cancer, it should be noted that individuals who consume a diet high in red and processed meat typically also consume large amounts of foods such as butter, potatoes, refined grains, and high-fat dairy, all components of a westernized diet [60]. Thus red and processed meat intake might not be solely responsible for higher cancer risk. Additionally, meat intake is usually correlated with higher energy intakes [61,62] and obesity [63], so residual confounding may be present. Research aimed at understanding how foods and nutrients interact to promote or prevent carcinogenesis may provide a better understanding of potential etiological pathways and may explain some of the heterogeneity of published results.

## Next Steps in Research

Further knowledge would be gained from research examining differences in particular subtypes of specific cancers. For example, different histologies or cancer subsites, such as estrogen-receptor-negative breast cancers or cardia gastric cancers, may be more strongly associated with dietary risk or preventive factors. Similarly, risk variation according to specific genotypes at polymorphic sites, for instance in genes involved in the metabolism of carcinogenic compounds in meat, may add further to our understanding of the role of meat consumption in cancer risk.

In addition to investigating intakes of food items or groups (i.e., red or processed meat), future research should also examine particular nutrients within meats (e.g., iron) or carcinogenic components (e.g., heterocyclic amines, nitrosamines) that are created as a result of certain cooking techniques, particularly among the rarer and less studied cancers. Other factors, such as animal raising and feeding practices (exogenous sex steroids are used in farm-raised animals in the US and banned in the farming industry in the European Union), may also contribute to cancer risk [64,65]. Few studies have examined these practices in their analyses, which may explain some of the inconsistency in results across studies.

## Conclusion

In summary, red and processed meat intake appears to be positively associated with risk of cancer of the colon and rectum, esophagus, liver, lung, and pancreas in a new, large US cohort study of 500,000 men and women. However, this study provided little support for an association with other cancer sites. Current dietary guidelines recommend selecting meats that are lean, low-fat, or fat-free [66], thus promoting limited consumption of red and processed meats. Overall, the strongest risk factors for cancer in the US are smoking and obesity [67]. However, understanding the complex interaction of diet with smoking and obesity, and how specific foods and nutrients are metabolized, may provide further clues into the etiology and, most importantly, the prevention of cancer.

## Abbreviations

AARP: formerly known as the American Association of Retired Persons
FFQ: food frequency questionnaire
NIH: National Institutes of Health
